# Effects of Salinity, Temperature, and Polyethylene Glycol on the Seed Germination of Sunflower (*Helianthus annuus* L.)

**DOI:** 10.1155/2014/170418

**Published:** 2014-12-28

**Authors:** Zhihui Luan, Moxin Xiao, Daowei Zhou, Hongxiang Zhang, Yu Tian, Yi Wu, Bo Guan, Yantao Song

**Affiliations:** ^1^Institute of Grassland Science, Key Laboratory of Vegetation Ecology, Ministry of Education, Northeast Normal University, Changchun 130024, China; ^2^Biology Department, Tonghua Normal University, Tonghua 134002, China; ^3^College of Automation, Harbin Engineering University, Harbin 150001, China; ^4^Northeast Institute of Geography and Agroecology, Chinese Academy of Sciences, Jilin 130102, China; ^5^College of Animal Science and Technology, Jilin Agricultural University, Changchun 130118, China; ^6^Educational Institute of Jilin Province, Jilin 130022, China; ^7^Yantai Institute of Coastal Zone Research, Chinese Academy of Sciences, Yantai 264003, China; ^8^College of Environment and Resources, Dalian Nationalities University, Dalian 116600, China

## Abstract

Salinization has severe influences on agriculture in the whole world. The main aims of this work were to evaluate osmotic effect and ion effect of NaCl on seed germination of three sunflower (*Helianthus annuus* L.) cultivars interacting with three alternating temperature regimes and to select the most salt tolerant cultivars to plant in the saline region. Seeds were germinated in the isotonic NaCl and polyethylene glycol (PEG) solutions of −0.45, −0.90, −1.34, −1.79, and −2.24 MPa at 10 : 20, 15 : 25, and 20 : 30°C temperature regimes. Both NaCl and PEG inhibited germination, but the effects of NaCl were less as compared to that of PEG, which means that adverse effects of PEG on germination were due to osmotic effect rather than specific ion accumulation. For the three cultivars, higher germination occurred at 10 : 20°C in NaCl treatments and at 20 : 30°C in the isotonic PEG treatments. Among the three cultivars, Sandaomei (SDM) is the most tolerant to salt and PEG stress.

## 1. Introduction

Seed germination is a crucial phase in the life cycle of plants [1], which determines the successful establishment of seedlings and subsequent growth. In this stage, plants are more sensitive to environmental stress than other growth and development stages. Several abiotic factors such as water, temperature, light, and salts that regulate seed germination interact in the soil interface [2]. These factors may act as stress leading to injury and even death of the plant in extreme cases [3]. Among these, however, salt is the main factor which limits seed germination and seedling establishment of plants growing in saline soil of arid and semiarid regions [4, 5]. In these regions, subsequent growth and final yield of crop plants are decreased when the salt level is very high.

Seeds of plant often germinate best under nonsaline conditions and their germination decreases with increase in salinity level [2, 6, 7]. Salinity stress can affect seed germination by creating an external osmotic potential that prevents water uptake or due to the ion toxic effects [8–11]. Ionic effects may be distinguished from osmotic effects by comparing the effects of salt solutions and isotonic solutions of an inert osmotic medium such as polyethylene glycol (PEG) that cannot penetrate into the cell wall. Inhibition of germination in PEG-treated seeds is attributed to osmotic effects, and any difference in germination of salt-treated seed, relative to PEG-treated seeds, is attributed to ionic effects [12]. Some studies have shown that the inhibitory effect of sodium chloride (NaCl) on seed germination was more severe than that of isoosmotic PEG, as in* Atriplex prostrata* and* Atriplex patula* [13] and* Aristida adscensionis* and* Artemisia ordosica* [14]. However, we found that Na^+^ had positive effect that promoted seed germination of barley in previous study [15]. A better understanding of how crop species respond to salt stress facilitates breeding work and other work that can increase crop production.

Sunflower belonging to the family Asteraceae is the world's fourth largest oilseed crop [16, 17]. And it is one of the most important economic crops in the oilseed production in China; also sunflower seeds are esculent and officinal [18]. With improvement of the living standard, sunflower has become more and more important in producing plant oil, especially in condition of controlled transgenic soybean. Thus, knowing the responses of sunflower to salt stress facilitates its development in this area. There are many researches on crop's responses to salt, such as soybean [19], rice [20], and wheat [21], but sunflower has little information on responses to salinity during seed germination.

Temperature can interact with salinity to affect seed germination. Although, higher salinity may inhibit germination, the detrimental effect of salinity is generally reduced at optimal germination temperatures. For example, in* Polygonum aviculare* [7] and* Sarcobatus vermiculatus* [22], decreased germination was noted at supraoptimal temperatures. However, the detrimental effects of salinity are more severe at lower temperatures for other species such as* Allenrolfea occidentalis* [23] and* Aeluropus lagopoides* [9]. In other species, the detrimental effect of salinity is severe both above and below the optimum, such as* Urochondra setulosa* [24] or* Sporobolus ioclados* [25]. Salinity-temperature interactions may have significant ecological implications in terms of time of germination under field conditions [2].

Thus, the aim of the present study was as follows: (a) to determine the germination response of sunflower seeds to NaCl solutions; (b) to differentiate the osmotic effect from ion toxic effect through comparison of NaCl with the metabolically inactive osmotic agent PEG; (c) to evaluate the effects of salinity/PEG, temperature, cultivars, and in particular the interactions between these factors on germination percentage and germination rate in sunflower seeds; and (d) to determine the most tolerant sunflower cultivar that will adapt to the arid or semiarid regions.

## 2. Materials and Methods

### 2.1. Seed Collection and Plant Cultivation

Three sunflower (*Helianthus annuus* L.) cultivars Sandaomei (SDM), Daxin (DX), and Longkui (LK) were used as seed materials in this study. They are all widely planted cultivars in the study region. Seeds of SDM and DX were obtained from a saline soil in Jilin Province (44.45°N, 123.45°E), northeast of China in October 2012, and seeds of LK were obtained from Institute of Crop Breeding, Department of Agricultural Science, Heilongjiang Province, in September 2012. Seeds were decorticated and achenes were stored at room temperature (23 ± 2°C) before use.

Germination experiments were conducted in March 2013 in three growth chambers (HPG-400, Harbin, China) with temperatures of 10 : 20, 15 : 25, and 20 : 30°C (dark : light). Seeds were germinated under different osmotic potentials induced by two osmotica, NaCl, and polyethylene glycol (PEG) at −0.45, −0.90, −1.34, −1.79, and −2.24 MPa. Distilled water was used as control. Seeds were germinated in twofold of filter paper placed in Petri dishes (diameter 12 cm) with 15 mL of test solution. The dishes were sealed with parafilm to prevent evaporation. Four replicates of 25 seeds were used for each treatment. Germination was recorded every day for 7 days. Seeds were considered to have germinated when the radicle emerged (1 mm).

The rate of germination was calculated using a modified Timson's index of germination velocity (IGV) = ∑*G*/*t*, where *G* is the percentage of seed germinated at 1 day interval and *t* is the total germination period [26]. The maximum value possible using this index with our data was 100 (700/7). Higher values represent a more rapid germination.

### 2.2. Statistical Analysis

Germination data was transformed (arcsine) before statistical analysis. A four-way analysis of variance (ANOVA) was carried out to test effects of main factors (temperature, cultivar, water potential, and osmoticum) and their interactions on the final germination percentage and rate of germination. A LSD test was used to determine least significant range between means (*P* < 0.05). These data were analyzed using SPSS (version 11.5, SPSS Inc., Chicago, Illinois, USA). The regression analysis between germination rate and osmotic potential was carried out using Sigma Plot (version 8.0, Systat Software Inc., Richmond, California, USA).

## 3. Results

### 3.1. Germination Time Courses and Final Germination Percentage

**Figure 1:**
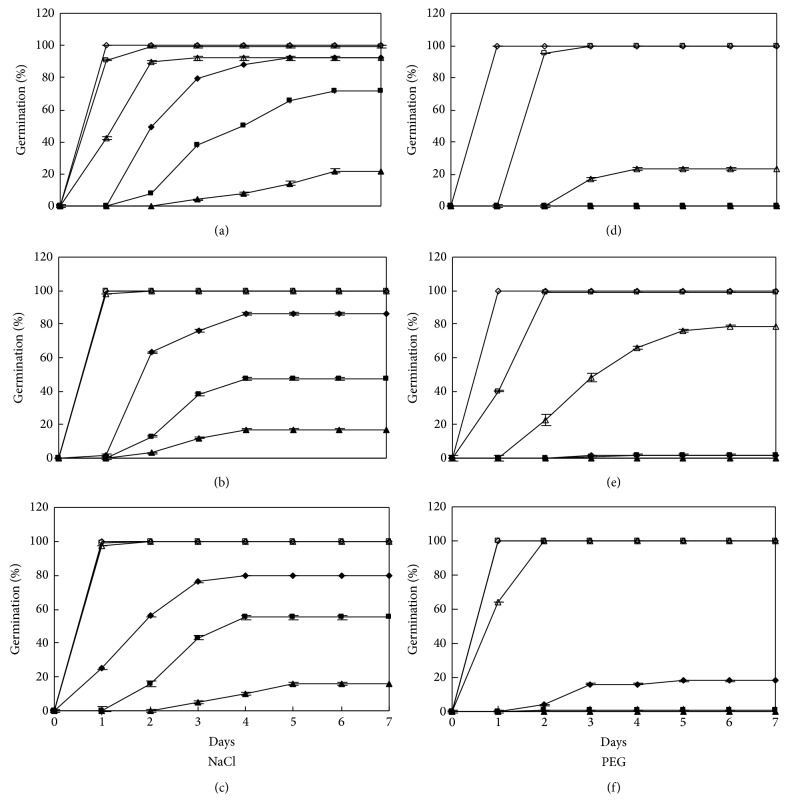
Germination time courses of DX in NaCl and PEG solutions at alternating temperature regimes. 0 (⋄), −0.45 (□), −0.90 (▵), −1.34 (◆), −1.79 (■), and −2.24 (▲) MPa; 10 : 20 (a, d), 15 : 25 (b, e), and 20 : 30°C (c, f). Bars indicate LSD at *P* = 0.05.

A four-way ANOVA indicated significant (*P* < 0.05) individual effects of temperature, cultivar, water potential, osmoticum (NaCl/PEG), and their interactions on germination percentage and rate of germination (Table 1). For cultivar DX, germination approached 100% in higher water potentials induced by NaCl solutions (−0.45 and −0.90 MPa) at all three alternating temperatures, the same as that in distilled water (Figures 1(a), 1(b), and 1(c)). Germination in lower water potentials was delayed for one or two days and decreased, with 47% in −1.79 and 17% in −2.24 MPa NaCl solutions at 15 : 25°C. In PEG treatments, all seeds germinated in −0.45 MPa solutions, but few seeds germinated below −0.90 and none in −2.24 MPa PEG solutions (Figures 1(d), 1(e), and 1(f)). Among the three temperatures, germination was best at 10 : 20°C for NaCl treatments and was significantly higher at 20 : 30°C for PEG treatments (*P* < 0.05, Figures 4(a) and 4(d)).

**Figure 2:**
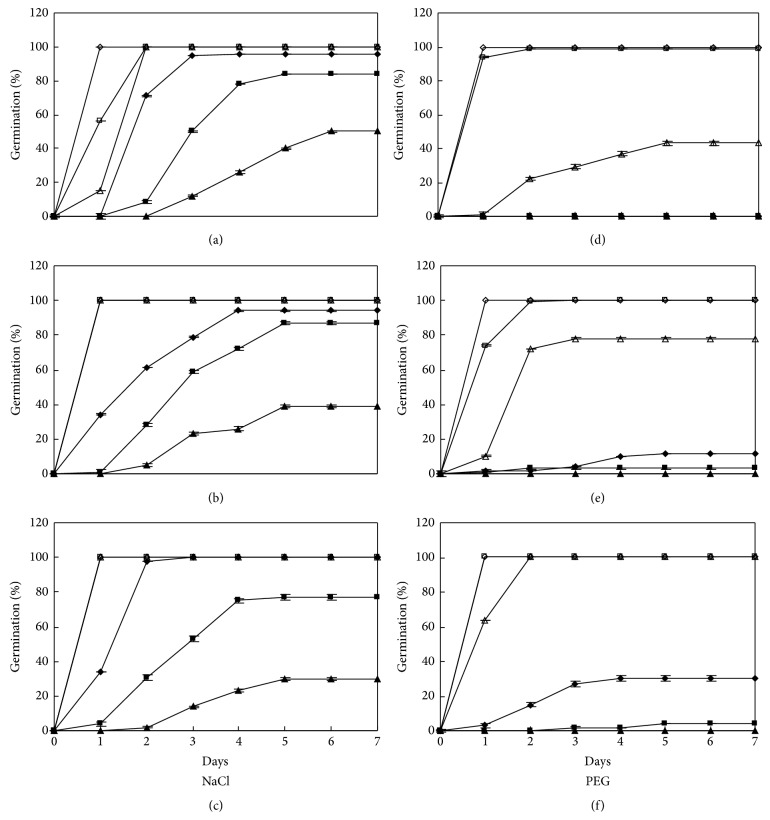
Germination time courses of SDM in NaCl and PEG solutions at alternating temperature regimes. See Figure 1 for symbols.

For cultivar SDM, germination was almost 100% in −1.34 MPa or higher water potential NaCl and slightly lower in −1.79 MPa NaCl solutions at all three temperatures. At 10 : 20°C, 50% seeds germinated in −2.24 MPa NaCl solutions with two-day delay (Figures 2(a), 2(b), 2(c), and 4(b)). The trend for PEG treatments was similar to that of cultivar DX with best germination at 20:30°C (Figures 2(d), 2(e), 2(f), and 4(e)).

**Figure 3:**
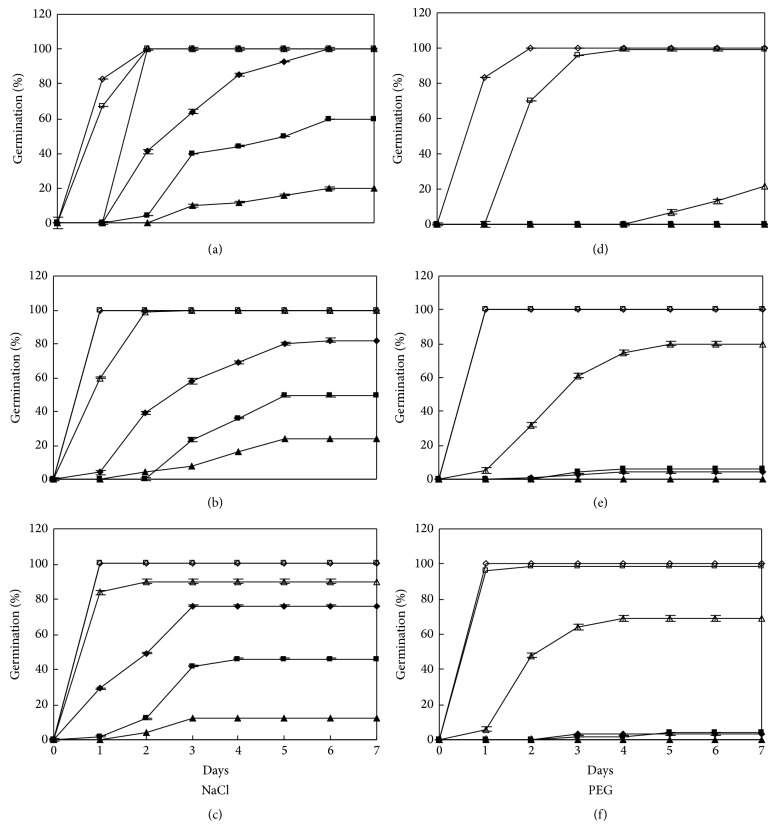
Germination time courses of LK in NaCl and PEG solutions at alternating temperature regimes. See Figure 1 for symbols.

**Figure 4:**
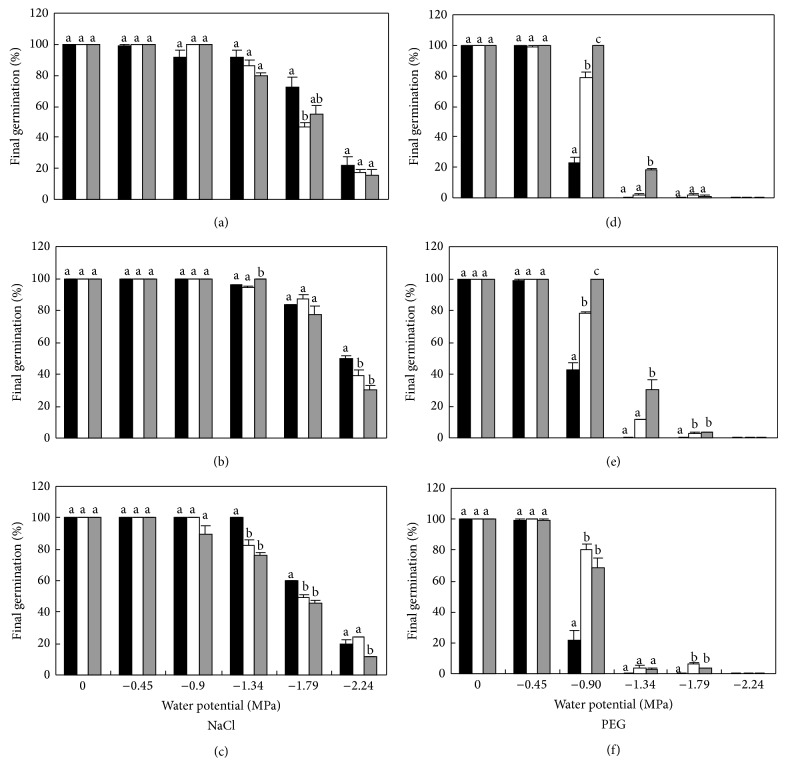
Final germination percentage of DX (a and d), SDM (b and e), and LK (c and f) in NaCl and PEG solutions at alternating temperature regimes. 10 : 20 (black bar), 15 : 25 (white bar), and 20 : 30°C (grey bar).

For cultivar LK, best germination was obtained at 10 : 20°C in NaCl and 15 : 25°C in PEG solutions (Figures 3, 4(c), and 4(f)). Among the three cultivars, final germination percentage of SDM was the highest in all water potential treatments at all three temperatures.

### 3.2. The Relation between Rate of Germination and the Water Potential

**Figure 5:**
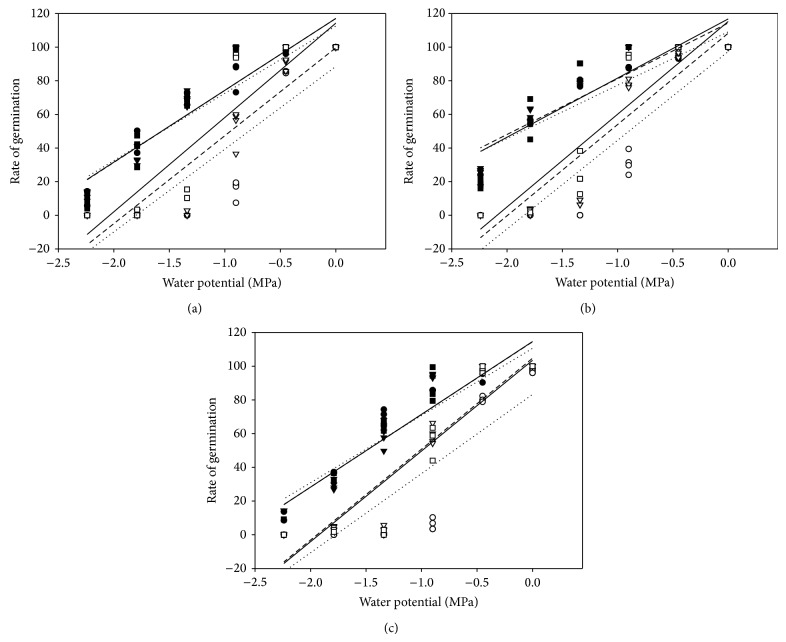
The relationship between rate of germination and the water potential under different temperature regimes in DX (a), SDM (b), and LK (c). Dotted line: 10–20°C NaCl (∙) and PEG (∘); short dash line: 15–25°C NaCl (▼) and PEG (▽); solid line: 20–30°C NaCl (■) and PEG (□).

Although germination approached 100% in higher water potentials induced by NaCl and PEG solutions, rate of germination was the highest in distilled water, decreased slightly in −0.45 and −0.90 MPa treatments, and decreased sharply as the water potential further decreased at all temperatures (Figure 5). Seeds germinated more rapidly in NaCl than in isotonic PEG and it decreased more dramatically in PEG than in NaCl with the decreasing water potential at all temperatures for all cultivars. The slope values of the PEG regression lines were all greater than the corresponding NaCl regression lines (Table 2). As the temperature increased, all cultivars exhibited increasing rate of germination irrespective of NaCl or PEG treatments, but the differences between temperatures were not significant for NaCl treatments (*P* < 0.05). For cultivar LK, rate of germination in PEG treatments at 15 : 25°C was slightly higher than that at 20 : 30°C (Figure 5(c)), which can also be seen from the parameters of the linear equations (Table 2). Of the three cultivars, SDM germinated most rapidly, and LK germinated most slowly in all temperature regimes.

## 4. Discussion

Seed germination of cultivar SDM was 100% or nearly at above −1.34 MPa NaCl solutions at all three temperatures and 50% in −2.24 MPa (500 mM) NaCl solutions at 10 : 20°C (Figures 2(a), 2(b), 2(c), and 4(b)). A halophytic perennial grass* A. lagopoides* in Pakistan germinated by 30% in 500 mM NaCl at optimum temperature of 20 : 30°C [9] and only 6% seeds of* Arthrocnemum macrostachyum*, a perennial halophytic shrub typical of Mediterranean salt marshes, germinated in 3% (510 mM) NaCl solutions at 20 : 30°C. Flowers [27] also pointed out that most crop plants will not grow in high concentrations of salt and only halophytes grow in concentrations of NaCl higher than 400 mM. These indicate that sunflower is a salt tolerant crop species, as stated by Qiu et al. [28].

NaCl and PEG both affected seed germination of sunflower severely; PEG had a greater inhibitory effect than NaCl for the three cultivars, especially in lower water potentials (−1.79 and −2.24 MPa). These results agree with the statements that NaCl had a less effect on the germination and seedling growth of cowpea than PEG [10] and seed germination was severely diminished by water stress induced by mannitol in sugar beet [29]. Ungar [30] also reported that inorganic ions are not inhibitory compared to mannitol and polyethylene glycol (PEG) in several halophytes and seeds are mainly affected by osmotic stress rather than specific ion toxicities [15].

In this study, in higher water potentials (−0.45 MPa, NaCl, and PEG), the germination was similar to the control and there were no significant differences (*P* < 0.05) between the two osmotica, which means the osmotic effect plays the main role. In water potentials between −0.9 and −2.24 MPa, ions of NaCl have a positive role: the ions in the cells could be used as osmolytes to maintain cellular osmotic potential lower than that of the ambient environment, allowing water entry into the cells [31]. The higher rate of germination and higher final germination in NaCl than in the isotonic PEG could be explained by the uptake of Na^+^ and Cl^−^ ions by the seed, maintaining a water potential gradient allowing water uptake during seed germination [15, 32]. The Na^+^ concentration results of SDM can provide part of the evidence (see Table S1 in Supplementary Material available online at http://dx.doi.org/10.1155/2014/170418). As the water potential decreased beyond −2.24 MPa, the toxic effect appeared, because the germination decreased to zero. The physiological regulatory mechanism of sunflower in salinity conditions would be studied in further experiments.

The four-way ANOVA indicated significant (*P* < 0.05) effects of temperature, cultivar, water potential, and osmoticum and their interactions on germination percentage and rate of germination (Table 1). This means that salinity and PEG affect seed germination significantly, and these effects change following varying temperature and different cultivars. For the three cultivars, higher germination occurred at lower alternating temperature (10 : 20°C) in the same water potential induced by NaCl. Decrease in germination at high temperatures may be correlated with increased evaporation of moisture, causing an increase in the salt content by capillary movement [33], and with a general lack of activation of metabolic processes and decrease in the levels of activity of different enzymes [34]. In contrast, higher germination was found at higher alternating temperature (20 : 30°C for cultivars DX and SDM; 15 : 25°C for cultivar LK) in the same water potential induced by PEG. One reason may be that high temperatures enhance the permeability of cellular membrane; thus more water can enter into it.

Genetic variability of different cultivars within a species offers a valuable tool for breeding work. In this study, the investigated cultivars showed different germination responses to PEG and salt stress, as well as the varying temperature regime. Cultivar SDM was the most tolerant to salt and PEG stress. DX was more tolerant than LK at higher (20 : 30°C) or lower (10 : 20°C) temperature and the opposite was observed at average temperature (15 : 25°C). LK also appeared to germinate more rapidly at 15 : 25°C than the other two alternating temperatures. This may be because cultivar LK is from Heilongjiang Province, which is in the north of Jilin Province and the annual temperature there is a little lower than Jilin Province. Thus, the optimum temperature for cultivar LK is lower.

Western Jilin Province (44.45°N, 123.45°E) in China belongs to the Northeast Farming-Grazing Interlocking District and arid and semiarid region, which was once a productive region but now has serious problems of salinization due to human activity frequently destroying the ecosystem equilibrium [35]. The average salt content of this area is 0.7 to 1% [36], which is equal to 120 to 171 mM NaCl. In arid or semiarid region, salinity effect is the most important factor that reduces the yield of crops. Sunflower, an annual herbaceous species in Compositae family, is the fourth worldwide economic oil plant species [16]. The crop yield is greatly dependent on the germination responses of the seeds. According to our experiments, sunflower can germinate in very high NaCl concentrations (500 mM) at all the selected alternating temperature regimes. The average salt content of this area is only 170 mM NaCl. Approximately half of the seeds can germinate in high salinity (500 mM) at optimum temperature (10 : 20°C) for cultivar SDM, which is the best among the three selected cultivars. The result can also provide some information for screening or breeding tolerant plant in future researches.

## Supplementary Material

Supplementary Table: The K^+^ concentrations of the early seedling in the isotonic PEG and NaCl solutions were similar, while the Na^+^ concentrations of the early seedling in NaCl solutions were dramatically greater than the PEG solutions with the same water potentials. This implies that Na^+^ can enter the seed and act positive roles in seed germination.

## Figures and Tables

**Table 1:** Results of four-way ANOVA of temperature (T), cultivar (C), water potential (P), and osmoticum (O) effects on final germination percentage and germination rate. All values are significant at *P* < 0.05.

Source	Final germination		Rate of germination	
	df	*F*	df	*F*
T	2	25.687	2	500.872
C	2	99.358	2	268.618
P	5	4664.443	5	10904.450
O	1	4102.480	1	5474.449
T × C	4	25.470	4	18.141
T × P	10	57.067	10	158.493
C × P	10	12.648	10	34.008
T × O	2	132.034	2	65.006
C × O	2	24.164	2	10.306
P × O	5	604.907	5	675.314
T × C × P	20	16.805	20	20.295
T × C × O	4	6.864	4	25.000
T × P × O	10	40.342	10	24.857
C × P × O	10	15.079	10	21.723
T × C × P × O	20	4.043	20	8.722

**Table 2:** The linear regression equations of rate of germination and the water potential under different temperature regimes. All regressions are significant at *P* < 0.05.

C	T (°C)	NaCl		PEG	
DX	10–20	*Y* = 112.564 + 40.024*x*	*R* ^2^ = 0.9127	*Y* = 88.535 + 49.202*x*	*R* ^2^ = 0.7880
	15–25	*Y* = 116.973 + 42.683*x*	*R* ^2^ = 0.8689	*Y* = 99.604 + 52.283*x*	*R* ^2^ = 0.8781
	20–30	*Y* = 117.049 + 42.539*x*	*R* ^2^ = 0.8688	*Y* = 114.237 + 56.079*x*	*R* ^2^ = 0.8287

SDM	10–20	*Y* = 109.054 + 31.745*x*	*R* ^2^ = 0.8870	*Y* = 97.2856 + 52.721*x*	*R* ^2^ = 0.8213
	15–25	*Y* = 114.293 + 33.127*x*	*R* ^2^ = 0.8291	*Y* = 108.189 + 54.250*x*	*R* ^2^ = 0.8666
	20–30	*Y* = 116.753 + 35.048*x*	*R* ^2^ = 0.7655	*Y* = 115.201 + 55.133*x*	*R* ^2^ = 0.9261

LK	10–20	*Y* = 110.565 + 39.854*x*	*R* ^2^ = 0.9106	*Y* = 83.197 + 46.923*x*	*R* ^2^ = 0.7439
	15–25	*Y* = 114.434 + 43.096*x*	*R* ^2^ = 0.9084	*Y* = 104.756 + 53.800*x*	*R* ^2^ = 0.9337
	20–30	*Y* = 114.608 + 43.103*x*	*R* ^2^ = 0.9186	*Y* = 103.52 + 53.738*x*	*R* ^2^ = 0.9326
