# Supplementary material for: Effects of Salinity, Temperature, and Polyethylene Glycol on the Seed Germination of Sunflower (Helianthus annuus L.)
Source: ScientificWorldJournal. 2014 Dec 28;2014:170418. doi: 10.1155/2014/170418 (PMC4295597; doi:10.1155/2014/170418)
Supplement: Supplementary file 1 — Supplementary Table: The K+ concentrations of the early seedling in the isotonic PEG and NaCl solutions were similar, while the Na+ concentrations of the early seedling in NaCl solutions were dramatically greater than the PEG solutions with the same water potentials. This implies that Na+ can enter the seed and act positive roles in seed germination. [file 170418.f1.doc]

Table S1 Na^+^, K^+^ concentrations (mg g^-1^ dry weight) and Na^+^/K^+^ ratio in seedlings of SDM in the isotonic PEG and NaCl solutions at 25/15^o^C.

| Water potential | Osmotica | Na^+^ | K^+^ | Na^+^/K^+^ |
| --- | --- | --- | --- | --- |
| 0 MPa | Distilled water | 1.056 | 8.609 | 0.123 |
| -0.45 MPa | PEG | 0.859 | 9.852 | 0.087 |
|  | NaCl | 13.384 | 9.838 | 1.360 |
| -0.90 MPa | PEG | 0.962 | 10.181 | 0.095 |
|  | NaCl | 10.319 | 10.399 | 0.992 |
